# Prevalence of Postoperative Infection after Tooth Extraction: A Retrospective Study

**DOI:** 10.1155/2021/6664311

**Published:** 2021-06-08

**Authors:** Elaine Kueh Yue Yi, Annabelle Lai Siew Ying, Mandakini Mohan, Rohit Kunnath Menon

**Affiliations:** ^1^School of Dentistry, International Medical University, Kuala Lumpur 57000, Malaysia; ^2^Clinical Dentistry, School of Dentistry, International Medical University, Kuala Lumpur 57000, Malaysia

## Abstract

The aim of the study was to identify the postoperative infection rates after tooth extraction in a university dental clinic and to identify the factors associated with an increased risk for postoperative infection. A retrospective study of case records of patients who underwent tooth extractions at the International Medical University's Oral Health Centre (IMU-OHC) over a span of 6 years was conducted. Data on demography, patient-related factors, and treatment-related factors were extracted from the case records. A binary logistic regression analysis was performed to assess the odds ratio of a patient having a postoperative infection or not, comparing it with each variable. A total of 1821 extractions, including simple and complex extractions, were performed over 6 years. Only 25 (1.4%) of the cases were reported to have a postoperative infection. The complexity of the extraction was the only variable that significantly affected the occurrence of postoperative infection after extraction; more complex extractions were reported with higher rates of infection (binary logistic regression, OR = 2.03, *p* = 0.004). None of the other factors, including antibiotic prescription, had a significant influence on the occurrence of postoperative infection. The prevalence of postoperative infection after dental extractions was low in IMU-OHC, and prescribing antibiotics had no added advantage in the prevention of postoperative infection.

## 1. Introduction

Tooth extraction is a procedure that is routinely carried out in dental practices worldwide. The main indications of these extractions include dental caries, periodontal problems, and pericoronitis associated with impacted teeth [1]. However, this conventional procedure may be associated with certain postoperative complications [2]. It is reported that one of the major complications following wisdom tooth extractions is infection, which may manifest as swelling, pain, abscess, fever, or a dry socket [3]. According to a Cochrane review of randomized controlled trials, the risk of postoperative infection after a third molar extraction in young patients who are physically fit is approximately 10%. However, the risk is increased up to 25% in patients with a low immune system prior to the extraction [4].

Even though some clinicians prescribe antibiotics to prevent postoperative infections after tooth extractions in their dental practice, the issue still remains a controversy in clinical practice up to this day as the possibility of patients acquiring an infection after extraction may be contributed by many factors. The use of antibiotics, the patient's gender, the patient's age, presence of systemic disease, smoking, the complexity of extraction, length of surgery, surgical technique, and surgical experience are some of the factors influencing the occurrence of infection after extraction [5–8]. Studies have shown mixed results regarding the effects of antibiotic usage after extractions, leading to an unsettled decision about the necessity of antibiotics postextraction. While antibiotics have been reportedly found to be successful in preventing infections after third molar extractions in some studies [9–11], there are other studies that opposed the usage of antibiotics with the aim of preventing postextraction infections [4, 12–18].

The efficacy of different antibiotics in lowering the risk of postoperative infections after third molar extractions has been reported, but they fail to provide a consensus regarding the type of antibiotic to be used and the appropriateness of antibiotic prescription [19]. Furthermore, a low infection rate (<5%) after third molar surgeries and the pressing issue of antibiotic resistance worldwide discourage the routine use of antibiotics if it is unnecessary [11]. A clear understanding of the effectiveness of antibiotics in the prevention of postoperative infection and the factors that contribute to infection can help dental practitioners carry out a more evidence-based approach in their practice.

Retrospective analyses of properly documented health records have shown to provide unique advantages in assessing outcomes with a low incidence, identifying multiple outcomes, and reducing the cost and time of a study [20]. The present study encompassed a 6-year retrospective follow-up analysis of case records of patients who have undergone tooth extraction. The aim was to evaluate the frequency of postoperative infection following tooth extractions that were performed between 01/01/2013 and 31/05/2019 at International Medical University's Oral Health Centre (IMU-OHC), to estimate the incidence of postoperative infection and to identify the factors associated with an increased incidence of postoperative infection.

## 2. Materials and Methods

Ethical approval for the study was obtained from the International Medical University Joint Committee on Research and Ethics.

A retrospective study of case records was conducted, involving all patients who have undergone tooth extractions at the IMU-OHC between 01/01/2013 and 31/05/2019. All case records that have been documented in the clinic's electronic health record system (OPENDENT) as tooth extractions were documented under the following treatment codes (EXTR001, EXTR002, EXTR003, EXTR005, EXTR007, EXTR008, and D7140). Extractions performed by both dental students or dentists were included.

To identify the postoperative infection rate, all records with the following treatment codes between 01/01/2013 and 31/05/2019 were examined in the study: EXTR001 as basic extraction of incisors and canines, EXTR002 as basic extraction of premolars, EXTR003 as basic extraction of molars, EXTR005 as complex extraction involving a surgical extraction, EXTR007 as complex extraction involving a routine surgery, EXTR008 as complex extraction involving a complex surgery such as deep impaction, and D7140 as extraction of an erupted tooth or exposed root.

Two investigators individually performed data collection after identifying the case records based on the treatment codes in OPENDENT, as stated previously. The data that were extracted from the case records included the patient's age (on the day of the extraction), gender, indication for extraction, type of operator, medical history, the complexity of extraction, antibiotic prescription, type of antibiotic given, postoperative infection, and type of postoperative infection (if any). Interoperator agreement on the coding for the indication of extractions was assessed by comparing the entries of both investigators and resolving any differences by discussion and agreement on the coding.

In addition, the indications for extractions were grouped into different categories (Table 1).

Postoperative infection was recorded as a clinical diagnosis of purulent discharge from the socket, pain with swelling, and increasing local swelling with or without suppuration. The antibiotic prescription guideline followed in IMU-OHC was in accordance with Malaysia's National Antibiotic Guideline (2014). Patients who needed additional treatment due to reinfection or persistent inflammation were also recorded. All records of extraction within the stated period were selected; however, any missed data were recorded as well. The data in the records were analysed accordingly.

The odds ratio for postoperative infection before and after the adjustment for confounding factors was investigated using R Version 4.1.0 employing binary logistic regression analysis using the function “glm ()”. The binary outcomes of infection = 1 and no infection = 0 were employed as the dependant variable. *p* < 0.05 was regarded as statistically significant. The independent variables were age, sex, complexity, operator, indication for extraction, and antibiotic prescription.

## 3. Results

A total of 1821 patient cases, which were categorized under various extraction treatment codes in the clinic's electronic health record system (OPENDENT), were analysed from 01/01/2013 to 31/05/2019.  Table 2 shows the total number of patients who underwent extractions, the number of patients with and without postoperative infection, and the variables that may influence the presence of postoperative infection after extractions.

Furthermore, as described in Table 3, the most commonly encountered postoperative infection in the patients was pain with swelling (12 cases), followed by 11 cases of pain and only 2 cases that had increasing local swelling with suppuration. Other postoperative infection types, such as purulent discharge and increasing local swelling without suppuration, as defined in our study, were not encountered.

The description of the prescribed antibiotics is provided in Table 4.

Table 5 presents the results of the binomial logistic regression analysis (Table 5). Among the variables for which data were extracted, only one factor “complexity of extractions” was associated with a significantly increased risk of postoperative infection (binomial logistic regression, OR = 2.03, *p* = 0.004). A simple extraction reduced the log odds for a postoperative infection by 2.03 times as compared to a complex extraction. None of the other factors had a significant influence on the odds of having a postoperative infection after extraction.

## 4. Discussion

According to the records, only 1.4% (25 patients) were reported to have postoperative infection after tooth extraction, whereas 31.8% (579 patients) had no complications after extraction and 66.8% (1217 patients) had no records of a follow-up appointment for problems related to the tooth extraction and hence were coded as having no infections. Twelve cases were from simple extractions, whereas 13 cases were from complex extractions. This is consistent with a prospective study, which concluded that the rate of postoperative infection such as infection in the alveolar bone, dry socket, and postoperative pain is low in routine extractions of erupted teeth [21]. Furthermore, a recent 5-year retrospective study reported that the infection rates after third molar extractions were minimal [11]. However, more than half of the extraction patient cases had no records of a follow-up, amounting to up to 66.8% of the total extraction cases. Thus, it is possible that some of these patients may have had a postoperative infection but did not return to IMU-OHC for treatment. Nevertheless, taking into consideration the overall low rates of postoperative infection after tooth extractions both in our study and in other reported studies, the need for antibiotics prescription for the purpose of preventing infection after tooth extractions is arguable.

Generally, antibiotic prescription after a dental extraction was not found to be routine practice in IMU-OHC as only 12.4% of the patients were prescribed antibiotics. Furthermore, only 1% of the patients who did not have an antibiotic prescription had a postoperative infection, while 3.6% of the patients who were given an antibiotic prescription presented with an infection after extraction. Some studies do not recommend the use of antibiotics in the prevention of postoperative infections after extractions, as their effects in reducing the risk of infection are not significant [4, 12–18]. However, success in using antibiotics to prevent postoperative infections after third molar extractions has also been reported [9, 10]. Nevertheless, considering the overall low incidence of postoperative infection in our study (1.4%), the low incidence of postoperative infection without antibiotic prescription (1%), and the occurrence of infection after extraction despite the prescription of antibiotics (3.6%), the prescription of antibiotics after extractions still remains disputable.

Furthermore, antibiotics should be prescribed with caution as antibiotic resistance is a global problem that has become a threat in the prevention and treatment of various infections [11]. In our study, the most popular antibiotic that was prescribed after extraction was amoxicillin in 61.6% of the patients who received antibiotics. Among the 8 patients who had a postoperative infection despite being prescribed antibiotics, all of them were prescribed amoxicillin or with one of its combinations; 5 were prescribed amoxicillin; 2 were prescribed amoxicillin and metronidazole; 1 was prescribed prophylactic amoxicillin. The common usage of amoxicillin demonstrates the preference for dentists to prescribe this antibiotic for preventing postoperative infections after extractions [11, 22–27]. However, a recent study reported the ineffectiveness of frequently used antibiotics such as amoxicillin in the prevention of postoperative infections after extractions, possibly due to antibiotic resistance [11].

Patients younger than 30 years of age had the highest incidence of postoperative infection (3.5%), while those in the age groups of 30–60 years and over 60 years recorded a 1.2% and 0.4% incidence of postoperative infection, respectively. This is in contradiction with previous clinical research, which reported that advanced age and higher comorbidity increase the risk of complications after extractions [28]. However, some studies indicated that the peak age range for a higher incidence of dry socket is approximately 20 to 40 years of age [29]. This may be due to third molar extraction surgeries being mostly done in young adults.

Among the 25 cases that presented with a postoperative infection, 22 cases (88%) were done by dental students, whereas only 3 cases (12%) were done by dentists and none by oral surgeons. As compared to a dentist and an oral surgeon, the operation time may be longer for students, thus indirectly increasing the risk of postoperative infection after extraction. It is noted that prolonged procedural time is an important risk factor for the occurrence of postoperative complications after oral surgery [30].

Extraction due to pericoronitis was the indication that had the highest incidence of postoperative infection (13.3%). This may be due to the presence of an existing infection before the extraction. A preexisting infection and pathology have been known to be associated with an increased risk for postoperative inflammatory complications following third molar surgeries [13, 28].

Additionally, we found that both genders had almost equal incidence of postoperative infection in this study. However, research that was done previously reported that the occurrence of a dry socket in women is eight times more than in men [31]. Moreover, patients who were in good health and patients who had existing medical illnesses such as hypertension or diabetes had the same ratio of postoperative infection incidence. Although significant medical history has shown to increase the risk of infection [13, 29], we considered the possibility that the medically compromised patients in our study had their medical conditions under control on the day of their extraction, thus presenting as complication-free during their review appointment.

Among the variables for which data were extracted, only one factor “complexity of extractions” was associated with a significantly increased risk of postoperative infection (OR = 2.03, *p* = 0.004). For the remaining factors, age, sex, operator, and the indication for the extraction or antibiotic treatment, we could not find a significant correlation with the occurrence of postoperative infection (*p* > 0.05). This may be due to the increased difficulty of the procedure, such as the need for a flap incision, bone removal, and tooth-sectioning. Several reports stated that lengthier surgeries tend to cause more painful sockets [5, 32–34]. However, other studies reported that only the factor of age was significant, in which the occurrence of infection after dental extractions was highest in older patients [11, 35]. This contrast may be due to the difference in the parameter of the types of extraction analysed, in which our study covered all types of extractions, whereas the 2016 study only focused on third molar surgeries (complex extractions).

## 5. Conclusion

In this 6-year retrospective case record study, the prevalence of postoperative infection after tooth extractions was reported to be low (1.4%) and no considerable advantage in prescribing antibiotics for the prevention of postoperative infection after tooth extractions was noted. Furthermore, among the factors that were analysed in this study, only the complexity of the extraction was found to have a significant impact on the occurrence of postoperative infection after dental extractions. With the issue of antibiotic resistance in mind, dental clinicians should prescribe antibiotics with discretion, and if antibiotics are indicated, the type of antibiotics should be prescribed judiciously via an evidence-based approach.

## Figures and Tables

**Table 1:** Indications for tooth extraction.

Category	Description
Periodontal disease (PD)	Teeth extracted due to poor periodontal prognosis
Pericoronitis (PC)	Teeth extracted due to inflammation of the gingiva surrounding the crown of partially erupted tooth
Pulpal pathology (C)	Teeth extracted due to pulpal pathology as a sequela of dental caries, such as irreversible pulpitis, apical periodontitis, and apical abscess
Orthodontic reasons (O)	Teeth extracted for fixed or removable orthodontic therapy
Adjacent tooth pathology (A)	Extraction of asymptomatic teeth due to dental caries, food impaction, or periodontal disease on an adjacent tooth
Elective extractions (E)	Extractions of asymptomatic teeth that were performed due to fear of future complications; such as an impacted third molar that may cause future pathological problems to an adjacent tooth
Retained deciduous teeth (D)	Extraction of asymptomatic deciduous teeth
Others (OX)	Extractions that cannot be categorized any other categories such as removal of supernumerary teeth or vertical tooth fracture, prosthodontic extractions of supraerupted or severely tilted teeth, and removal of teeth as a result of dental trauma

**Table 2:** Patient and treatment variables showing the number of infection cases reported for each variable.

Variable	Total	Infection	No infection
Teeth	1821	25	1796

*Age*			
<30	314	11	303
30–60	968	12	956
>60	539	2	537

*Sex*			
Female	941	13	928
Male	880	12	868

*Indication*			
Periodontal	586	4	582
Pericoronitis	15	2	13
Pulpal pathology	521	7	514
Orthodontics	33	1	32
Adjacent tooth pathology	17	0	17
Fear of future complication	158	8	150
Deciduous	11	0	11
Others	480	3	477

*Operator*			
Student	1675	22	1653
Dental surgeon	146	3	143

*Medical history*			
No relevant history	1487	21	1466
Hypertension/diabetes/heart	334	4	330

*Complexity*			
Simple	1594	12	1582
Complex	227	13	214

*Antibiotic*			
No	1592	17	1575
Yes	229	8	221

**Table 3:** The types of postoperative infection for the 25 postoperative infection cases.

Types of postoperative infection	No. of cases
Increasing local swelling with suppuration	2
Pain	11
Pain with swelling	12

**Table 4:** Antibiotics prescribed for tooth extraction.

Types of antibiotics	Total	Infection	No infection
Amoxicillin	141	5	136
Amoxicillin + metronidazole	36	2	34
Augmentin	24	0	24
Metronidazole	12	0	12
Others	16	1	15

**Table 5:** Binomial logistic regression with postoperative infection as the dependent variable.

Variable	Estimate	Standard error	*Z* value	*p* value
Sex (M)	4.12	4.166*e* + 01	0.099	0.921
Age (>60)	−9.36	9.320 + 01	−1.005	0.314
Age (30–60)	2.02	5.549 + 01	0.037	0.970
Complexity (si)	−2.03	7.134 + 01	−2.848	0.004^*∗*^
Operator (S)	4.59	6.841 + 01	0.671	0.502
Indication (C)	1.44	1.524 + 03	0.010	0.992
Indication (D)	−1.42	2.486 + 03	0.000	0.999
Indication (E)	1.42	1.524 + 03	0.009	0.992
Indication (O)	1.51	1.524 + 03	0.010	0.992
Indication (OX)	1.40	1.524 + 03	0.009	0.992
Indication (PC)	1.59	1.524 + 03	0.010	0.991
Indication (PD)	1.44	1.524 + 03	0.009	0.992
Antibiotic prescription (Y)	2.77	5.473 + 01	0.507	0.612

M: male; Si: simple; S: student: C: pulpal pathology; D: deciduous; E: elective; O: orthodontic; OX: others; PC: pericoronitis; PD: periodontal disease.

## Data Availability

The data used to support the findings of this study are available from the corresponding author upon request.
